# Non-Contact Respiration Monitoring and Body Movements Detection for Sleep Using Thermal Imaging

**DOI:** 10.3390/s20216307

**Published:** 2020-11-05

**Authors:** Prasara Jakkaew, Takao Onoye

**Affiliations:** 1 Information Systems Synthesis Laboratory, Department of Information Systems Engineering, Graduate School of Information Science and Technology, Osaka University, 1-5 Yamadaoka, Suita, Osaka 565-0871, Japan; onoye@ist.osaka-u.ac.jp; 2 School of Information Technology, Mae Fah Luang University, 333-1 Thasud, Muang, Chiang Rai 57100, Thailand

**Keywords:** respiration monitoring, non-contact monitoring, body movements detection, thermal imaging, natural sleep environments

## Abstract

Monitoring of respiration and body movements during sleep is a part of screening sleep disorders related to health status. Nowadays, thermal-based methods are presented to monitor the sleeping person without any sensors attached to the body to protect privacy. A non-contact respiration monitoring based on thermal videos requires visible facial landmarks like nostril and mouth. The limitation of these techniques is the failure of face detection while sleeping with a fixed camera position. This study presents the non-contact respiration monitoring approach that does not require facial landmark visibility under the natural sleep environment, which implies an uncontrolled sleep posture, darkness, and subjects covered with a blanket. The automatic region of interest (ROI) extraction by temperature detection and breathing motion detection is based on image processing integrated to obtain the respiration signals. A signal processing technique was used to estimate respiration and body movements information from a sequence of thermal video. The proposed approach has been tested on 16 volunteers, for which video recordings were carried out by themselves. The participants were also asked to wear the Go Direct respiratory belt for capturing reference data. The result revealed that our proposed measuring respiratory rate obtains root mean square error (RMSE) of 1.82±0.75 bpm. The advantage of this approach lies in its simplicity and accessibility to serve users who require monitoring the respiration during sleep without direct contact by themselves.

## 1. Introduction

The respiratory rate is one of the critical vital signs that indicate health problems. The respiratory system is the gas exchange process to take in oxygen and expel carbon dioxide, as breathing moves air in and out of the lungs. The frequency of breaths is defined as a respiratory rate (RR) that is usually measured by counting the number of breaths a person takes per minute. A clinical staff member can count the number of times the chest moves up and down for a full minute. In general, the regular respiratory rate for healthy individuals is 12–20 bpm [1]. A change as little as three to five bpm may indicate a change in the patient’s condition [2]. A patient suffering a severe adverse event on the general wards, such as a cardiac arrest or ICU admission, shows an increase in RR of up until 24 h before the severe events with high specificity [3]. For that reason, respiration monitoring should be performed continuously for a long time without impressing the patient’s burden.

Owing to the fact that humans spend almost 30% of the time in sleeping, the respiration monitoring during sleep, which accurately reflects one’s health condition is an appropriate and reasonable option. It is generally known that poor sleep significantly affects work productivity, cognitive performance, and overall life quality. Sleep monitoring can detect sleep disorders associated with cardiovascular disease, including heart failure, hypertension, and increased arrhythmia [4]. The breathing patterns during sleeping are utilized to identify the sleep disorder as sleep apnea, including obstructive sleep apnea (OSA), central sleep apnea, and complex sleep apnea syndrome. Sleep apnea is a cessation of the airflow that occurs when breathing repeatedly stops and starts during sleep, resulting in decreased oxygen flow to the brain and the rest of the body. Sleep apnea is generally characterized by cessation of breath for at least 10 s during sleep [5]. The well-known index used to indicate the severity of sleep apnea is Apnea–Hypopnoea Index (AHI), which counts the number of apnea events per hour. Harvard Medical School [6] classifies the severity of OSA as:

- None/Minimal: AHI < 5 per hour
- Mild: AHI ≥ 5, but < 15 per hour
- Moderate: AHI ≥ 15, but < 30 per hour
- Severe: AHI ≥ 30 per hour

Besides, sleep monitoring can detect periodic limb movement disorder (PLMD), which is repetitive cramping or jerking of the legs during sleep. Patients with PLMD may suffer from daytime sleepiness, daytime fatigue, trouble falling asleep at night, and difficulty staying asleep throughout the night [7]. Usually, patients with PLMD are unaware of their leg movements unless their bed partner tells them. It is also reported that movements are repetitive and rhythmic and occur every 20–40 s [8].

It has been demanded for long years to develop a novel technology to monitor respiratory and body movements without disturbing human sleeping. In recent years, non-contact respiration monitoring solutions have been proposed, like camera imaging [9], thermal imaging [10], and microwave Doppler radar [11]. As for camera imaging, a simple camera like a CCD camera, webcam, digital camera, or smartphone camera is used to detect the respiratory rate from the breathing motion, whereas for thermal imaging, a thermal camera or infrared camera is used to detect the temperature changes due to breaths. Respiratory rate often relies on visual observation of chest movement at periodic intervals [1]; a small movement that is hard to see with the naked eye. A motion magnification technique was proposed to magnify a baby’s respiratory chest movements from a digital video camera [12]. Several researchers have analyzed video and image sequences to detect breath motions and extract vital signs in sleeping positions. For example, Nakajima et al. and Frigola et al. apply an optical flow technique to image data from a CCD camera and a remote TV camera, respectively [13,14]. Wiesner et al. monitor respiratory by tracking the motion of a fiducial marker placed on the patient’s abdomen with a single webcam [15]. However, there are still some limitations of disturbing the natural sleep environment, such as visible light and privacy issues.

Thermal imaging is a rapidly evolving technology, now turning up in hospitals, airports, and even smartphones. Respiratory can be monitored through thermal imaging [16,17,18,19,20,21]. Thermal imaging cameras rely on microelectromechanical sensors to produce an image from heat; the human body stands out from the surrounding field because it gives off more heat. The thermal image-based method has an advantage under conditions of varying illumination and can reduce privacy issues. Previously, several approaches have been proposed to monitor respiration with a thermal camera by detecting the temperature change around the nostrils [18,21,22,23,24,25,26,27,28] or the airflow [19,20,29] in seated positions. They set the nose or the mouth as the region of interest (ROI) that can be defined manually or automatically by using anatomical features integrated with tracking algorithms [18,21,22,23,25]. They performed by simulated breathing following scenarios that the researcher designed, i.e., regular breathing, fast breathing, and hold breathing [18,21,24,25,26]. The excellent result showed when they took the experiments in a controlled room like temperature, humidity, and light. However, the nose detection during sleep is still unsuccessful at all monitoring times.

In the sleeping position, the thermal-based method is an effective technique to measure the nasal airflow patterns [16] and has been utilized to detect sleep apnea [30,31], analyze sleep activity [32], classify body posture [33] during sleep to assist the diagnosis of sleep disorders or evaluation of the quality of sleep. The studies using thermal imaging to monitor the respiration in sleeping position are reviewed in Table 1. Usman et al. [30] adopted thermal imaging to detect sleep apnea and study in a variety of breathing patterns. They used the Kanade–Lucas–Tomasi tracking algorithm to track the manual selected nose region. The result has shown that 16% of a subject’s head position did not allow correct identification of the region of interest at nostrils. Therefore, this method was only possible with minor head movement without changing position. The automatic ROI selection was used to locate the nostrils, the tip of the nose, and mouth area [31,34,35]. That ROI requires a tracking algorithm and works well without large head movement under a controlled environment. Abbas et al. [16] developed respiration monitoring for neonatal intensive care units by manually select the ROI around the nostrils of infant. Most techniques work well when the nose is clearly visible in the image. The measurement was not feasible when the nose is outside the camera’s field of view, a blanket blocks the nose, or the subject has large head movements. Recent works from Pereira et al. and Lorato et al. [36,37] detected the respiration signal without the use of anatomical features. They selected the ROI containing the respiration information by using the Signal Quality Index to analyze the ROIs. However, they took an experiment in a controlled environment with a short period that is not a real environment. Moreover, the motion artifacts are still a significant drawback of the proposed algorithm. It was suitable for monitoring infants in neonatal care who did not have large movements.

The present study aims to develop the measuring system capable of non-contact monitoring of respiration and body movements in natural sleep environments using a thermal video. The natural sleep environments imply an uncontrolled sleep posture, darkness, and covered subjects with a blanket. In the study, the different approaches based on temperature detection and motion detection were investigated to extract the respiration signal, and then the suitable approach is selected. Our main contributions of this paper are to present: (1) the respiration monitoring based on ROI detection combined with breathing motion, which does not require facial landmarks’ visibility; (2) body movement detection to estimate the numbers of movement during sleeping that affect sleep quality. We validated the proposed approach by comparing it with the signal obtained from the respiratory belt.

## 2. Proposed Method

**Figure 1:**
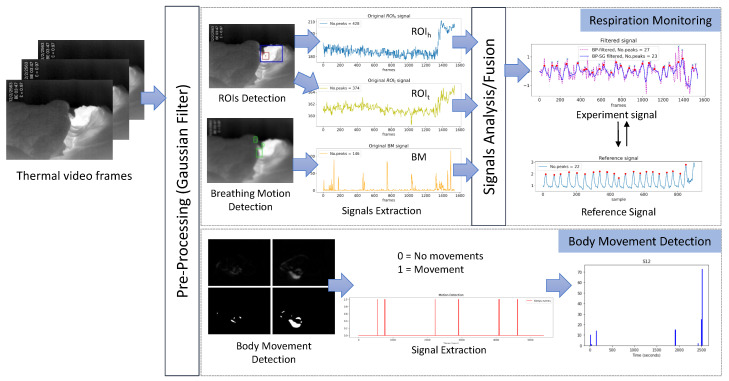
Proposed method.

In this section, the proposed method for respiration monitoring and body movements detection is described. An overview of the proposed method is depicted in Figure 1. The input of the proposed method is the thermal video obtained under darkness light. The Gaussian filter is applied to the input images as the pre-processing so as to remove noises from the input. The main part of the proposed method is composed of respiration monitoring and body movements detection, each of which utilizes image processing and signal processing techniques in order. Details of these processes will be written below.

### 2.1. Respiration Monitoring

The respiration monitoring method contains an automatic detection of ROIs by finding the highest temperature point and the largest portion of the high-temperature area and a breathing motion detection. The respiration signals extracted by automatic ROI detection and breathing motion detection are integrated. Then the signal processing is applied to calculate the respiratory rate.

#### 2.1.1. Automatic ROI Detection

We employ the ROI detection to limit the observation area, extracting important information to raise the accuracy of the respiratory estimation. Determining a suitable ROI position with proper size is also important. In the sleep monitoring environment, it is not easy to detect a face or nostrils as an ROI because of the uncontrolled sleep posture, and the fixed camera position may mean that they could make a face that does not appear in the camera view on some occasions. Besides, when the subject changes the sleep posture, ROI should be updated to the new location for which some research applied a tracking algorithm, as summarized in Section 1. The tracking algorithm works well with an apparent object, but sometimes fails to track the nose or mouth in a sleeping posture. In this work, we propose an ROI detection on the thermal image in a sleeping position that does not require a tracking algorithm. Two different ROI detections are considered: (1) The highest temperature point detection and (2) The largest portion of high-temperature area detection.

**Figure 2:**
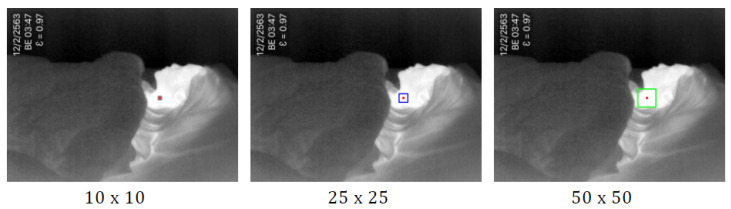
The sample of three different $ROI_{h}$ sizes as 10×10, 25×25, and 50×50 (red point indicates the highest temperature point).

(1) The highest temperature point is detected by using minMaxLoc. The minMaxLoc function is one of the OpenCV [38] libraries that returns minimum and maximum intensities found in an image with their (x,y) coordinates. It is assumed that the maximum pixel intensities of the thermal image refer to a human’s heat signature that is not covered by a blanket. The maximum pixel intensities found in the image correspond to the highest temperature of the body. We set the pixel to the center of the observation area. Then we draw a rectangle around the pixel, with the size of the square N×N pixels depending on original frame resolution. In [39], the authors compared the ROI size of 10×10, 25×25, 50×50, 100×100, and 150×150 pixels. They found that the size of the ROIs for respiratory rate estimation is usually smaller than that for heart rate estimation. Therefore, in this study, we consider the three different $ROI_{h}$ sizes as 10×10, 25×25, and 50×50, as shown in Figure 2. The result in empirical research has shown that the 50×50 pixels provided the highest accuracy in accordance with the original frame resolution of 640 × 480.
(2) The largest portion of the high-temperature area is detected by using the thresholding method. Thresholding is the method of segmenting the object from the background. The threshold image g(x,y) can be defined as (1) [40]:
  (1) $$g\left(x,y\right)=\left\{\begin{array}{ccc} 1 & if & I\left(x,y\right)\geq T_{R}\\ 0 & if & I\left(x,y\right)<T_{R} \end{array}\right.$$

This operation replaces each pixel values in an image with a white pixel if the pixel intensity I(x,y) is greater than or equal to the threshold value ($T_{R}$), assigned to a black pixel if the pixel intensity is less than $T_{R}$. In this work, the white pixels are considered to represent the human skin area.

To determine the threshold value $T_{R}$, we coordinated empirical research with varying the value among 128, 144, 160, 176, 192, 208 and 224. It was confirmed that the $T_{R}$ value 176 is the one that yielded the best results in all the performed tests.

**Figure 3:**
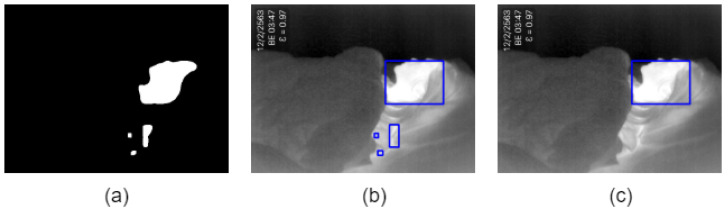
(**a**) All contours, (**b**) bounding rectangles around all contours, and (**c**) bounding rectangle around the most prominent contour.

Figure 3a shows the segmentation of the input image with the thresholding method. Then, we used the findContours function to find the location of white regions that return the outlines corresponding to each of the white blobs on the binary image. The bounding box is drawn around those contours (see Figure 3b). Finally, we find the most prominent contour and bounding box around that contour, as shown in Figure 3c. In this study, we selected the biggest box as $ROI_{t}$.

Then $ROI_{h}$ and $ROI_{t}$ are cropped for extract signals $\bar{S_{h}}\left(f\right)$ and $\bar{S_{t}}\left(f\right)$ by computing the average of pixel values (2) within each ROI of each frame, where S(x,y,f) is the pixel values of the thermal image at pixel (x,y) in the video frame *f*, $N_{*}$ is the vector of pixel coordinates in $ROI_{h}$ or $ROI_{t}$, and $n_{*}$ is its number.

(2) $$\bar{S_{*}}\left(f\right)=\frac{1}{n_{*}}\sum_{x,y\in N_{*}}S\left(x,y,f\right)$$

#### 2.1.2. Breathing Motion Detection

Breathing motion detection applies, in the background, a subtraction method for detecting the motion by calculating the difference between the current frame and previous frame. Specifically, absolute difference for all pixels between the current frame I(x,y,f) and the frame one second before I(x,y,f−s) is calculated (3):

(3) B(x,y,f)=|I(x,y,f)−I(x,y,f−s)|,

where *s* is the frame rate.

Then, we extract portions of moved area by using thresholding, erosion, and dilation operations. Parameters used in these operations are 5 for thresholding pixel value difference is less than 5 and 5×5 kernel for opening (i.e., erosion and dilation). Next, bounding boxes are determined by finding contours with filtering out small movement as noise. Finally, the number of bounding boxes are counted as the metric of breathing motion (BM).

#### 2.1.3. Respiration Signal Analysis

There are three respiration signals extracted by ROIs detection based on temperature detection and breathing motion detection. The following are the steps to estimate the respiration signals.

**Figure 4:**
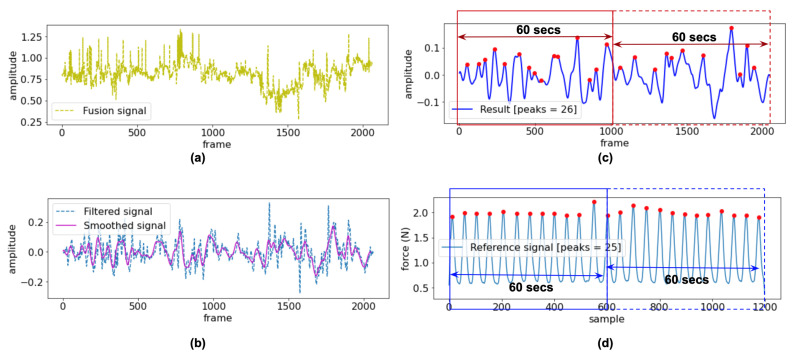
(**a**) Sample of fusion signal, (**b**) filtered and smoothed signals, (**c**) peak detection of experiment signal, and (**d**) peak detection of the reference signal.

(1) The respiration can be extracted by detecting the chest movements, the breaths airflow, and the temperature change around the nostrils. However, the detection in a specific method cannot be guaranteed in the sleep monitoring because of the fixed camera position, and an independent subject posture may make the region out from the camera view. In such a case, an alternative method for respiration detection is required. It is reasonable to assume that the respiration can be detected by blending the temperature change of ROIs and breathing motion. Therefore, we combine three signals by employing the root mean square (RMS) to calculate the average of the respiration signals as (4).
  (4) $$Respiration\;signals=\sqrt{{ROI_{t}}^{2}+{ROI_{h}}^{2}+BM^{2}}$$
(2) The 3rd order of Butterworth bandpass filter [41] with a lower cutoff frequency of 0.05 Hz and a higher cutoff frequency of 1.5 Hz was applied. The frequency bound is equivalent to 3–90 bpm, based on the typical RR for an adult person (12–20 bpm) and monitoring the abnormal RR that is less than 12 bpm and higher than 20 bpm.
(3) The Savitzky–Golay (SG) filter is a least-square polynomial filter that reduces noises while retaining the shape and height of waveform peaks [42]. Here, the SG filter was used to smooth the signal after the bandpass filter. The SG filter’s output increased the precision of the data without distorting the signal tendency. There are two parameters of the SG filter, including window length and the filter order, which closely relates to the performance of the filter. In this study, we tested the parameters and selected the optimal values to get the best-filtered signal, i.e., the window length of 51 and the polynomial order at 3rd were used. The result of SG filter still includes the small peaks, and thus a moving average is calculated to detect only the desired peaks and ignore small ones.
(4) The fusion signal in Figure 4a was smoothed by the SG filter and moving average (see Figure 4b), and then the number of peaks is counted. Figure 4c depicted the peaks detection of the experiment signal, followed by the peaks detection of the reference signal in Figure 4d, which are assumed to correspond to the number of breaths. The findpeaks function is used with adjusting the width as 10 based on empirical research.
(5) The number of peaks is calculated as breaths per minute (bpm) for each 60 s slice of input video (1020 samples at 17 fps) and was compared with the reference RR. For performance comparison, the accuracy of the RR estimation was tested using the RMSE defined as (5)
  (5) $$RMSE=\sqrt{\frac{1}{N}\sum_{i=1}^{N}{\left(x_{i}^{exp}-x_{i}^{ref}\right)}^{2}}$$
  where *N* is the total number of the slices, and $x_{i}^{exp}$ and $x_{i}^{ref}$ represent the experimented and reference RR values obtained for slice.

### 2.2. Body Movements Detection

This process aims to determine the more significant action than the respiration representing the big movement like limb movement, head movement, and full position change during sleep. First, an absolute difference image $B^{\prime }\left(x,y,f\right)$ between adjust two thermal images (I(x,y,f) and I(x,y,f−1)) is obtained by $B^{\prime }\left(x,y,f\right)=\left|I\left(x,y,f\right)-I\left(x,y,f-1\right)\right|$. Then, we binarize the difference image by thresholding method.

**Figure 5:**
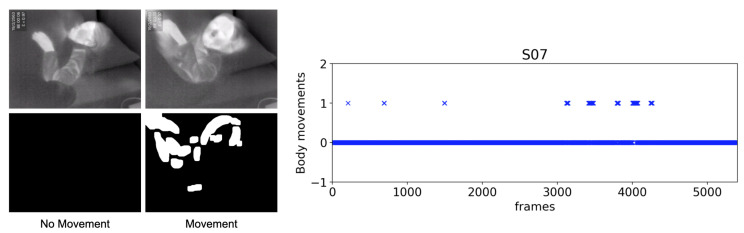
Sample output of the body movements detection.

In the same manner as the breathing motion detection, we extract portions of the moved area by erosion and dilation operations. While using 35 for thresholding in order to detect large body movements, the same parameters are used for erosion and dilation. Then, we apply the findContour function to examine whether those are a portion of moved area. If any contour is found, the body movement signal is set to 1. An example output of the body movement is shown in Figure 5, where the left panel is the movement detection, and the right panel plots the movement signal.

## 3. Experimental Results

This section analyzes the signal gathered in two experiments. Experiment (1) the respiration monitoring, and experiment (2) the body movements detection during sleep. The results were compared with the reference signals obtained by the Go Direct Respiration Belt.

### 3.1. Experimental Setup

We assessed the performance of our proposed non-contact monitoring of respiration and body movement detection under natural sleep environments. During the experiments, the thermal videos were captured using a portable thermal camera (Seek Thermal Compact PRO for iPhone) attached to a smartphone and fixed on a tripod in front of a participant located at approximately 100 cm. The camera was set at the proper position so that the upper body of a participant was apparent in the camera’s field of view. The Seek Thermal Compact PRO is a highly portable thermal imaging camera with a wide, 32-degree field of view. This thermal camera has a resolution of 640 × 480 pixels and detects infrared wavelengths in the spectral range of 7.5 to 14 Microns. The camera’s emissivity was set to 0.97, as this is suitable for human skin temperature measurement [43]. Besides, the videos were recorded at 17 frames per second (fps). The Go Direct Respiration Belt [44] was used as a reference to collect human respiratory effort and respiratory rate from a force sensor and an adjustable nylon strap around the chest during respiration. The measuring parameters were set to 10 samples/s, and the duration is approximately 5400 s.

**Figure 6:**
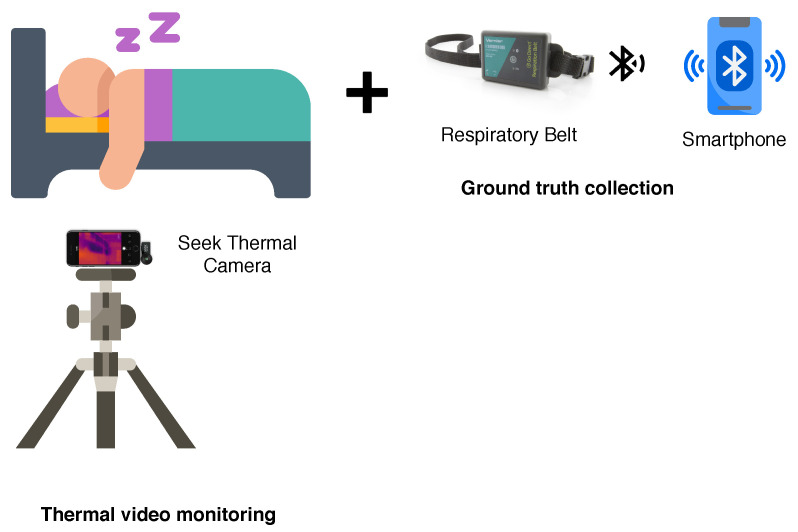
Data collection setup.

The data were collected on different days, from multiple camera positions with volunteers wearing different clothing. The experiments were conducted on real-life conditions, and volunteers were invited to record in their room while they were sleeping. They placed a respiratory belt around their ribs and mounted a thermal camera on a tripod by themselves before they go to bed. Figure 6 illustrates an environment setup. Sixteen healthy volunteers with ages between 25 years old and 37 years old (29.88±3.26 years old), ten females and six males, with height between 151 cm and 180 cm (162.63±7.37 cm), weight between 47 kg and 78 kg (57.38±9.28 kg) and body mass index (BMI) between 18.65 kg/m${}^{2}$ and 27.64 kg/m${}^{2}$ (21.64±2.98 kg/m${}^{2}$) volunteered for this experiment. Table 2 shows the participants’ data.

### 3.2. Respiratory Rate Estimation and Body Movements Detection

Table 3 contains the results obtained for all subjects, including respiratory rate estimation and body movement detection. The respiratory rate estimated by our proposed method was compared with the reference signal obtained by the respiratory belt. The RMSE was calculated by considering all the breaths in each signal collected during the experiment minute-by-minute. The average respiratory rate in the overall subjects is 14.78±1.93 bpm for the reference signal and 14.47±0.60 bpm for the proposed approach. The standard deviation of RMSE for the respiratory rate of all subjects is 0.75 bpm, and the average is 1.82 bpm. The small RMSE indicates that the proposed approach is robust for subjects variation. As for body movements detection, we counted the number of movement, the number of frames including body movement, and the total duration of body movements as summarized in Table 3.

**Figure 7:**
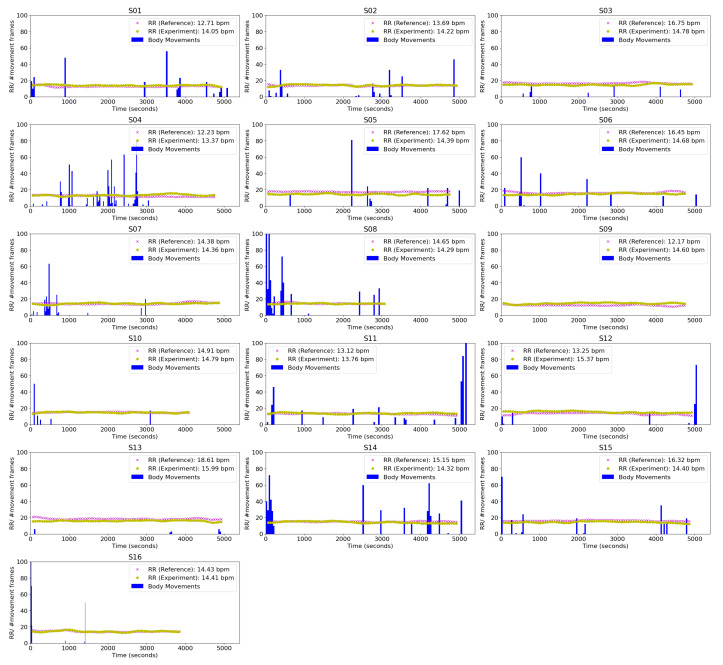
The result of the RMSE (respiratory rate) and body movements of S01–S16.

The histogram of the number of body movements in every 40 s is also calculated to check the symptoms of PLMD. A small movement like a limb or head movement while sleeping takes a short duration, while significant movements or position changes can take a long time. Therefore, we calculated the degree of body movements by dividing the movement period with the number of movements, which is assumed to be closely related to sleep quality. Figure 7 shows the respiratory rate and body movements of S01–S16. The blue bar represents an occurred body movement. Several times in the event, in the beginning, refer to difficulty falling asleep like subject 4 and subject 8. The reference and experiment’s respiratory rates were plotted ‘x’ and ‘o’, respectively. The blue column represents the histogram of body movements every 40 s. From this table, we can confirm that there was no regular and repetitive body movements for all subjects during experiments, which is the typical phenomenon of PLMD.

Our proposed approach provides respiration and body movements monitoring, which no existing thermal image based sleep monitoring system presents. We believe that body movement detection would characterize abnormal movements and behaviors during sleep, and is more comfortable for the users and completely unobtrusive.

## 4. Conclusions

This paper proposed an approach for non-contact respiration monitoring and body movements detection in a natural sleep environment using a thermal camera. The thermal camera can handle many viewing angles, which makes installation in the bedroom easy. We have to overcome specific challenges to acquire non-contact respiration data from participants in their natural sleep environment when the lights were turned off and they were covered by a blanket. The thermal video sleep monitoring can be performed in a dark environment to settle privacy concerns. The participants were asked to set up the system and perform a recording by themselves at their home. This approach aims to use for screening the irregular respiration before going to the hospital.

The proposed approach consists of automatically computing the ROIs that it can use to acquire the respiration signal and detecting the body movements of the participant by employing an image processing on the continuous thermal image. The signals were obtained in each frame process with normalizing and smoothing signals. Then, we computed the number of breathing and counted the number of body movements. The approach has been validated using a respiratory belt as a reference signal. We evaluate respiration monitoring performance and body movements detection in different rooms with 16 participants who have independent sleep postures. Our results show that the proposed approach successfully estimated that the RR was obtaining an RMSE of 1.82±0.75 bpm. The performed experiments confirmed that a thermal camera is easy to use for respiration monitoring and body movements during sleeping within various environments. In future work, we will focus on monitoring a patient who has irregular breathing. Another element of our work that could be improved is the automatic optimization of the thresholding value. The other limitations of the proposed method, such as a variation of room temperature, the type of bed cover, blanket, and night sweats (neck or face) in subjects, rapid eye movement (REM) stage, and heart rate, provide ideas for addressing these issues in future studies.

## Figures and Tables

**Table 1:** A research review of the thermal imaging-based method for respiration monitoring of sleeping position.

					ROI Localization		
Authors	Subjects	Exp Duration	Controlled Env	Simulated Breathing	Selection/ Detection	Area	Tracking
Usman et al. [30]	Adult	5 min	Yes	Yes	M	Nostrils	Yes
Fei et al. [31]	Adult	60 min	Yes	No	A-S	Nostrils	Yes
Al-Khalidi et al. [34]	Children	2 min	Yes	No	A-S	Tip of the nose	Yes
Hu et al. [35]	Adult	10 min	Yes	Yes	A-S	Nose, mouth	Yes
Abbas et al. [16]	Infant	2 min	Yes	No	M	Nostrils	No
Pereira et al. [36]	Infant	5 min	No	No	A-D	N/A	No
Lorato et al. [37]	Adult	2 min	Yes	Yes	A-D	N/A	No
Our proposed	Adult	60–90 min	No	No	A-D	N/A	No

M: Manually, A-S: Automatically Selection, A-D: Automatically Detection.

**Table 2:** Participants Data.

Subjects	Gender	Age (years)	Height (cm)	Weight (kg)	BMI (kg/m${}^{2}$)
S01	F	28	162	56	21.34
S02	F	36	167	52	18.65
S03	F	31	162	50	19.05
S04	F	29	163	53	19.95
S05	F	32	158	54	21.63
S06	M	25	161	70	27.01
S07	F	31	151	47	20.61
S08	F	29	160	50	19.53
S09	M	30	168	55	19.49
S10	F	28	159	58	22.94
S11	M	28	180	75	23.15
S12	M	26	169	58	20.31
S13	M	27	168	59	20.90
S14	M	29	168	78	27.64
S15	F	32	153	56	23.92
S16	F	37	153	47	20.08

**Table 3:** The result of respiratory rate estimation and body movements detection.

Subjects	Respiratory Rate (bpm)				Body Movements			
	Duration (s)	Reference	Experiment	RMSE	#Movements	#Frames	Duration (s)	Degree
S01	5371.05	12.71	14.05	1.56	14	269	15.69	1.12
S02	5397.54	13.69	14.22	1.11	15	199	11.65	0.78
S03	5379.37	16.75	14.78	2.20	7	63	3.69	0.53
S04	5192.31	12.23	13.37	2.00	35	642	37.86	1.08
S05	5212.78	17.62	14.39	3.32	9	200	11.72	1.30
S06	5200.51	16.45	14.48	2.23	9	214	12.53	1.39
S07	5332.39	14.38	14.36	1.47	16	218	12.80	0.80
S08	3495.39	14.65	14.29	1.18	15	749	43.48	2.90
S09	5407.22	12.17	14.60	2.68	0	3	0.17	0.00
S10	4520.26	14.91	14.79	0.75	5	91	5.33	1.07
S11	5346.70	13.12	13.76	1.25	16	417	24.42	1.53
S12	5361.45	13.25	15.37	2.35	7	140	8.20	1.17
S13	5399.74	18.61	15.99	2.79	5	20	1.17	0.23
S14	5380.52	15.15	14.32	1.49	16	535	31.14	1.95
S15	5315.23	16.32	14.40	1.99	12	225	13.19	1.10
S16	4287.12	14.43	14.41	0.72	6	250	14.51	2.42
Mean	5100.00	14.78	14.47	1.82				1.21
STD	537.63	1.93	0.60	0.75				0.74
